# Mutation Analysis of *TP53* Tumor Suppressor Gene in Colorectal Cancer in Patients from Iran (Kerman Province)

**Published:** 2012

**Authors:** Azadeh Lohrasbi Nejad, Mohammad Mehdi Yaghoobi

**Affiliations:** 1 *Department of Biotechnology, Research Institute for Environmental Sciences, International Centre for Science, High Technology and Environmental Sciences, Kerman, Iran *

**Keywords:** Colorectal cancer, Point mutation, tp35 tumor suppressor gene

## Abstract

**Objective(s):**

*P53* is an important tumor suppressor, which is mutated in later stages of many cancers and leads to resistance to chemotherapy. The aim of this study was to reveal mutations of *TP53* in colorectal cancer in Kerman province.

**Materials and Methods:**

A total of Forty-three colon cancer specimens as paraffin block or fresh tissues, which passed stage IIIA, were selected. Three exons 5, 7 and 8 of *P53* were amplified by PCR technique and sequenced directly.

**Results:**

The results showed two deletions at codon 140 and 142 in one tumor sample. GAT→AAT mutation at codon 184, and CGG→TGG mutation at codon 248 were seen in some tumor samples. Some mutations were also observed in middle of intron 7 in some tumor or normal tissues.

**Conclusion:**

Some of those patients with mutation in *P53* gene had metastasis in other organs. Therefore, genetic test before chemotherapy is helpful for successful treatment.

## Introduction

Colorectal cancer (CRC) is one of the commonest malignancies in the world (1). There are approximately 500,000 annual new cases of CRC worldwide (2). Excluding skin cancers, CRC is the third most common cancer diagnosed in men and the second leading cause of cancer-related deaths in the . The clinicians recorded that approximately 147,000 adults were diagnosed with CRC in the United States in 2009 (3). CRC is staged according to extent of tumor spreading to nearby lymph nodes and metastases to distant organs (liver or lungs). Staging is an important part of diagnosis, treatment planning, and predictions of long term survival (4). 

Tumorigenesis in CRC is a multi-step phenomenon, which is typified by a series of genetic events that lead to invasive malignant tumor from normal epithelium (4, 5). Mutation in some genes leads to an increased risk of CRC. Besides chromosomal instability, about seven distinct genetic changes are required for a normal cell to progress toward adenoma and eventually carcinoma (6, 7). Of those genes characterized to date, activation of the oncogene Kirsten-ras (K-ras) and inactivation of tumor suppressor gene *P53* are important determinants of tumor initiation and progression (7-9).

*TP53* is a principal mediator of a number of cellular functions, including growth arrest and apoptosis in response to DNA damage (10). It stops cell cycle in damaged cells until alteration is properly repaired, otherwise it initiate apoptosis cascade in damaged cells (11). If this guardian of genome becomes inactivated upon mutation, it could not execute its duty and more mutations will be accumulated in the cell and eventually lead to cancer development (11). In addition to suppressing cancer development, normal *P*53 gives sensitivity to chemotherapy and radiotherapy in tumour cells.

Human *P53* gene is composed of 11 exons and codes a protein with 393 amino acids. More than than 50% of human tumors and cancer cell lines have mutation in this gene. About 90% of all mutations occur in exons 5-8 that correspond to central DNA binding region of the protein (12). 

On the other hand, about 95% of CRC are adenocarcinoma and show a high frequency of *P53 *mutations when metastasized to other organs (13, 14). Interestingly, CRC is curable if detected early. However, with traditional diagnosis criteria majority of patients are not diagnosed until advanced stages of disease (13, 15). Studying molecular characteristic of *P53 *mutations and their functional consequences can help us in diagnosis, prognosis, and therapy of human cancers (16, 17). One study showed over-expression of mutant *P53* in 63% of patients with CRC (18). Cytoplasmic accumulation of abnormal *P53* is a bad prognostic factor for survival in CRC (19). 

In addition, patients with colorectal cancer show variable responses to standard treatment. Some mutations in this gene lead to resistance to 5-fluorouracil, oxaliplatin, irinotecan or other common chemotherapy drugs in CRC and lung cancer (20-23). Mutations in *P53 *also result in resistance to platinum-based chemotherapy in ovarian cancer (24). Irinotecan is the second line of chemotherapy for advanced stage colorectal cancer. *P53 *status of tumour affects sensitivity of CRC to irinotecan. *P53* wild type cells are more sensitive to irinotecan treatment compared to mutant *P53* (25). Therefore, expression of this marker should be monitored for better management of patients with advanced CRC. In such situation another drugs or protocols for treatment should be replaced. 

It is clear that pathological staging could not reveal *P53* mutations or their type. Therefore, molecular testing can predict a patient response to treatment and prognosticate its future. Hence, searching for mutations of *P53 *and their effects on efficiency of therapy is essential in management of cancers. In other words, an effective treatment can be planned after revealing *P53* mutations (13, 15, 20, 26). More than 3500 mutations in *P53* have been reported in CRC so far. The total identified mutations of *P53* have been compiled on http:// p53.free.fr/Database/ p53 database.html or other databases. Most of these mutations result in loss of function of the protein (12). 

To our knowledge, no comprehensive study has been done on mutations of *P53* in colorectal cancer in alls area of Iran. There is a report on *P53 *mutations in colorectal cancer from northern Iran (27). They detected *P53 *mutation in 44.4% of samples and found that they correlated directly with stage of cancer. Also, mutation types differed between proximal and distal CRC (27). In this research, we investigated *P53* mutations in specimens of CRC by polymerase chain reaction** (**PCR) and direct sequencing. The aim of this study was to determine *P53* mutations in colorectal samples and compare them with the other mutations in databases and utilize their potential for diagnosis and prognosis in the future. 

## terials and Methods

***Patients***

A total of eighty six specimens of colorectal carcinoma tissues as formalin fixed paraffin embedded (FFPE) blocks were obtained from pathology archive of public hospitals (Bahonar, Afzalipour, Aliebne-abitaleb and Hazrat-Fatema) or private laboratories (Dr Dabiri) in Kerman during 2002-2008. Tumors were staged and graded according to criteria of Tumor-Node-Metastasis (TNM system) classification (4). The study included only forty three samples that were in stage IIIA or later (with metastasis in at least two regional lymph nodes). Diagnostic slides from each case were reviewed to confirm carcinoma and identify tumor area in all cases. Six samples of fresh tumor were also obtained from patients undergoing surgery due to aggressive colorectal carcinoma upon informed consent. Patient’s clinical information was also retrieved from pathology records. Fresh samples were frozen in liquid nitrogen and stored at -80 °C until DNA isolation. Paired normal colon tissue from both archive and fresh samples were also collected for comparison. 

***DNA extraction***

Genomic DNA was prepared from peripheral blood, fresh tissues or paraffin- embedded tissue blocks with DNeasy kit (Qiagen) containing mini spin column according to manufacturer protocol with minor modification for paraffin blocks specimen. The tumor area in paraffin blocks was firstly isolated from surrounding tissue and minced with homogenizer. Paraffin was removed by xylene-ethanol treatment and samples were heated at 98 C for 15 min before adding proteinase K. The next steps of extraction were the same for all specimens as the company recommended. All precautions were considered to avoid contamination between normal and tumor samples of a patient or between different patient samples.

Quantity and quality of extracted DNA was surveyed by 260/280 spectrophotometer and agarose gel electrophoresis respectively.

***PCR***

Three oligonucleotide primer pairs (Table 1) were designed and used to amplify fragments of 158, 193 and 202 bp corresponding to all extent of exons 5, 7 and 8 of the human *TP53* gene. DNA concentration, cycling time and other amplification conditions were optimized for each primer pair on blood DNA.

The fragments were amplified in 25 µl reaction volume containing 100-600 ng genomic DNA; 0.3 µM of each primer; 200 µM dNTP mix; 5% DMSO; 1:2.5 U *Pwo *(Roche) and *Taq* polymerase (Cinnagen) respectively.

PCR was performed in the Mastercycler (Eppendorf) machine under the following conditions: 94^ ◦^C for 5 min, followed by 15 cycle of 94 ^◦^C for 30 sec, 55-60^ ◦^C for 30 sec, and 72 ^◦^C for 30 sec. Subsequently the extension time was increased 5 sec at each cycle and reached to 130 sec in the last thirty fifth cycle. Then 72 ^◦^C was applied for 5 min as final extension. Amplified fragments were separated on 1.5% agarose gel electrophoresis, stained with EtBr and photographed with G BOX HR (). 

Different exons and different specimen were extracted and amplified separately to prevent cross contamination.

***DNA sequencing***

Twenty µl of PCR products were run on agarose gel electrophoresis and recovered with Silica Bead DNA Gel Extraction Kit (Fermentas) according to company’s protocol. The recovered DNA was quantified by 260/280 spectrophotometer.

Recovered PCR products (2 ng/µl) were dissolved in 15 µl volumes and were sequenced using forward primer (Value Read) via Eurofins MWG Operon (). The results were analyzed in Chromas (Technelysium) and SeqScanner (Applied Biosystems) softwares. All sequences results (normal and tumor) were compared with the UMD *TP53* database at http://p53.free.fr/Database/p53_database.html to examine for the presence of new mutations.

## Results

**Figure 1:**
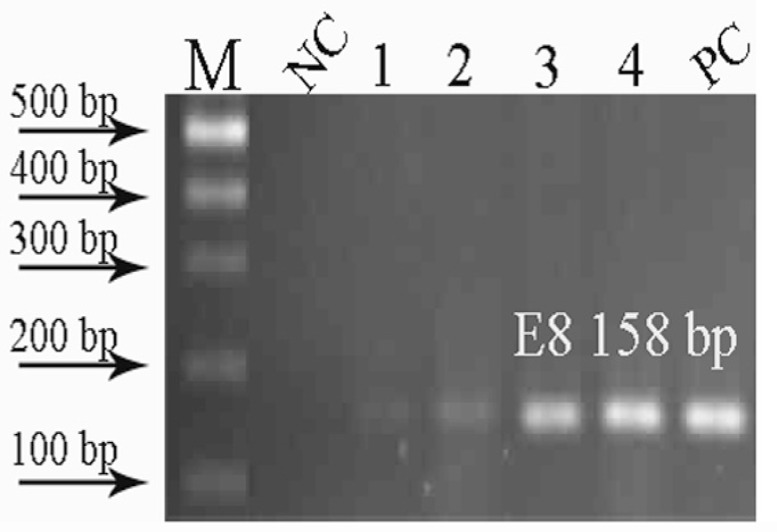
PCR products amplified by *Taq* Polymerase (1, 2) and *pwo* (3, 4) on agarose gel electrophoresis. NC=Negative control, PC=Positive control. As it is obvious the exon 8 segment is substantially amplified by *pwo* while weak band is seen when *Taq* polymerase is used in PCR reaction.

During sampling, either some specimens were not at stage IIIA or their diagnostic slides were not found. DNA was extracted from archival and fresh colorectal carcinoma samples, and three exons were amplified by PCR. Despite extensive attempts and optimization, certain archival samples did not provide DNA suitable for PCR. Consequently these samples were excluded and only those samples with a discrete band of amplified exons were selected for sequencing. The PCR results showed that *Pwo* polymerase was more strength than *Taq* polymerase on amplification of exons from FFPE samples (Figure 1). PCR products were recovered from agarose gels and sent for sequencing. Sequences of the exons were determined in both tumor and normal specimens from 11 patients (two fresh and nine archival samples). No mutations or polymorphisms were observed in fresh tissues, and all nucleotide changes including four point mutations were found in FFPE specimens. 

**Figure 2:**
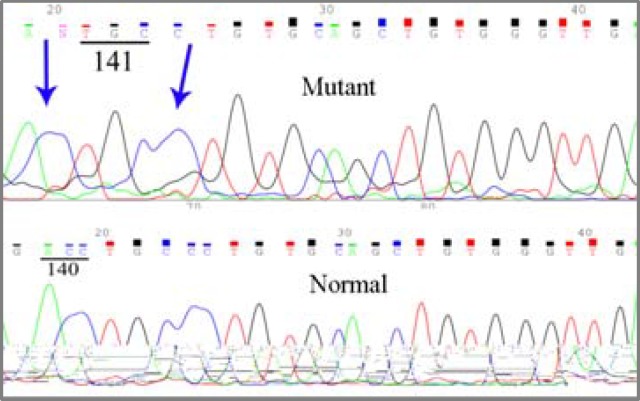
Sequencing result of tumor DNA of a patient shows deletion at codon 140 and 142. Normal tissue of the patient has normal sequence.

One tumor sample showed a double deletion: ACC→A∆C at codon 140 and CCT→∆CT at codon 142, while the corresponding normal tissue had a normal sequence (Figure 2). 

**Figure 3:**
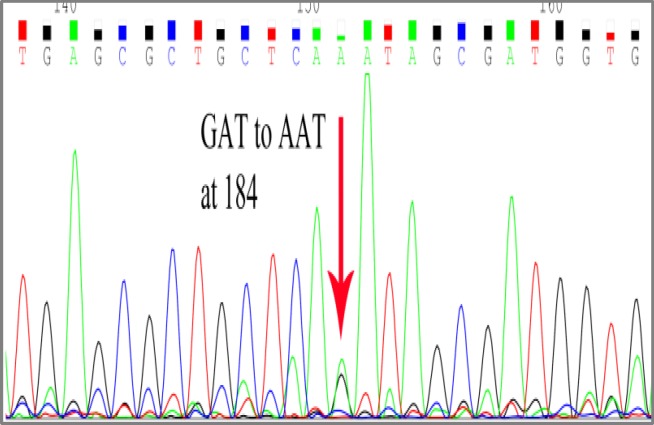
Sequencing of a tumor DNA shows GAT→AAT conversion at codon 184.

GAT→AAT mutation was observed in another tumor sample at codon 184 (Figure 3).

**Figure 4:**
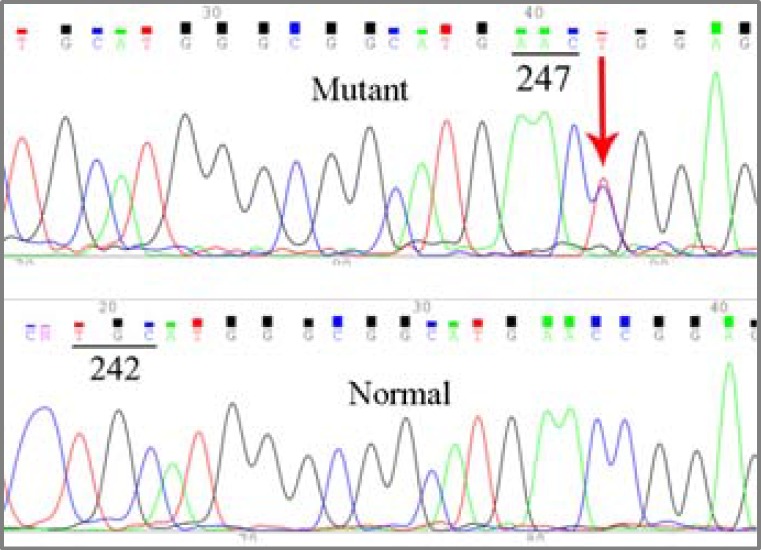
Sequencing result of two tumor DNAs show CGG→TGG conversion at codon 248.

Two tumors also showed a CGG→TGG mutation at codon 248 (Figure 4). 

Totally five mutations were identified: three missense mutations and two mutations leading to frameshift (Table 2). Normal patient sequences did not show any abnormalities or any of the above-mentioned mutations. 

**Figure 5:**
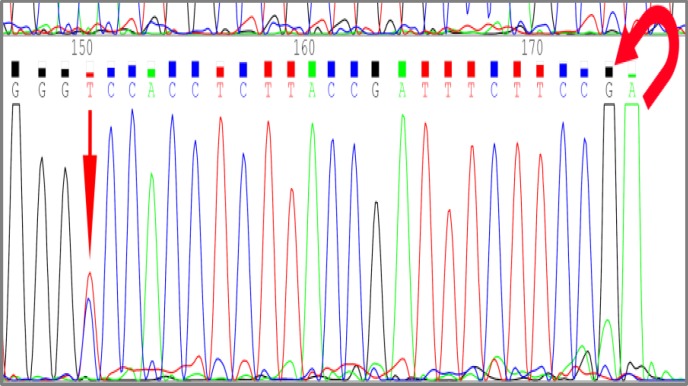
Insertion of G and substitution of T insteead of C in the middle of intron 7-8.

In addition, three tumors and two normal samples showed an insertion of a G nucleotide in the middle of intron 7. Of these samples one tumor and one normal sample were from the same patient. Moreover, a tumor sample had both a G insertion and a CCC→TCC mutation at the same intron (Figure 5). Interestingly normal tissue from this patient had neither of these polymorphisms.

## Discussion

Archival FFPE specimens are valuable resource for molecular genetics and cancer research. However, Extraction and amplification of DNA from these tissues often have little efficiency, because formalin damages DNA and reduces its quality (28, 29). Some researchers tried to optimize extraction and amplification of DNA from fixed tissues (28, 30). We recently optimized extraction of DNA from these samples and examined their amplification with different kinds of Polymerases. Our findings showed treating at 98 °C before extraction and amplifying with a mixture of *pwo* and *Taq* polymerases could enhance efficiency of PCR on FFPE specimen (31). With this protocol we have successfully amplified and sequenced FFPE tissue DNA. Four mutations were identified in our FFPE samples. Double deletion at codon 140 and 142 was seen in tumor tissue of a patient. These deletions lead to shift in reading frame and a premature stop codon is created at codons 169 or 173. Thus, a truncated protein lacking DNA binding domain is synthesized. Such deletions have been reported previously in lung (32), breast (33) and ovary (34) cancers, but they have never been reported in CRC. Thus, this is the first report of such mutation in colorectal cancer.

**Table 1:** The sequences of primers used for amplification of exons 5, 7 and 8 of *TP53* gene

	Sequence of primer	Length of PCR product
EXON 5	F: GTACTCCCCTGCCCTCAACA R: CTGCTCACCATCGCTATCTG	193 bp
EXON 7	F: GGCTCTGACTGTACCACCAT R: GGAAGAAATCGGTAAGAGG	202 bp
EXON 8	F:GGTAATCTACTGGGACGGAAC R: GCTTCTTGTCCTGCTTGCTT	158 bp

**Table 2:** The results of *P53* mutations found in colorectal cancer samples

Sample No	Codon (exon)	Wild-type codon	Mutant codon	Mutation type	Wild amino acid	Mutant amino acid
T25	184 (5)	GAT	AAT	Transition	Asp	Asn
T35	142 (5) 140 (5)	CCT ACC	∆CT A∆C	Frame shift	Pro Thr	
T45 , T46	248 (7)	CGG	TGG	Transition	Arg	Trp
T19, T35, T46	Intron 7-8		Insertion of G			
N35, N48	Intron 7-8		Insertion of G			
T19	Intron 7-8	CCC	TCC			

The second mutation that was observed in this research is GAT→AAT transition at codon 184, which put Asn instead of Asp in the protein chain. Such mutation has been identified in breast (35) and colorectal (8) and recently in liver (36) cancers. There is a report of an association between smoking and such mutation in lung cancer (37). Probably some exogenous carcinogens may induce such mutation in large intestine. How this codon is mutated (by endogenous or exogenous agents) and how it interrupts the normal function of *p53* protein need further functional study.

Another mutation that we found in two patients is CGG→TGG transition at CpG dinucleotide at codon 248. As we previously said the central DNA binding domain is target of 90% of *P53* mutations found in human cancers. The most frequent mutations in different cancers including colon cancer are situated at codon 175, 248 and 273 (12). A positive correlation between the frequencies of C:G→T:A transitions at CpG dinucleotide sites in *P53* and inducible nitric oxide synthase activity has been described in human colon cancer (38). Clinical files of the two patients showing mutation at codon 248 reported that both of them had metastatic cancer in their liver. 

In addition to the above mutations, some polymorphisms were also observed in intron 7-8. Obviously, such polymorphisms have no clear consequence on the protein function. G insertion in the middle of intron may be a hereditary polymorphism. Since both normal and tumor tissues of a patient showed it. 

We encountered some limitations in this research. Sampling was restricted to those that had passed stage IIIA, so we could not collect more samples. Furthermore, most samples were as FFPE, and a number of them did not give an amplifiable DNA. 

Some samples did not show any apparent mutation. That may be due to the fact that they had not really passed stage IIIA or they may had mutation in other exons except exons 5-8. Although, passing this stage did not guarantee that the *P53* gene was mutated. 

Analyzing mutation of *P53* in CRC in other regions of IRAN and in other prevalent cancers could be done to compile an inclusive data bank on mutation of *P53* in IRAN. We also suggest that pathology laboratories use fixatives other than formalin to preserve quality of DNA for future analysis by molecular methods such as PCR.

Studying relation between different kinds of mutation and possible chemotherapy resistance could also help us in design of a successful therapy plan. Besides, quantitative measurement of gene expression in different stages of CRC may disclose a suitable genetic marker for the diagnosis of cancer stages. 

## Conclusions

Although extraction and amplification of DNA from FFPE is laborious, with some alteration even old-age or low quality samples can be analyzed at molecular level. In this research we studied *P53* mutations in FFPE samples of colorectal cancer and we found some new mutations not previously reported in CRC. Considering *P53* mutation in cancer patients is critical in determining a successful treatment manner. The current chemotherapy or radiotherapy methods for cancer are completely dependent on *P53* function, because they induce the intrinsic pathway of apoptosis only when *P53* is normal (8). 
